# Supplementary figures and images for: Complex genetic patterns in human arise from a simple range-expansion model over continental landmasses
Source: PLoS One. 2018 Feb 21;13(2):e0192460. doi: 10.1371/journal.pone.0192460 (PMC5821356; doi:10.1371/journal.pone.0192460)

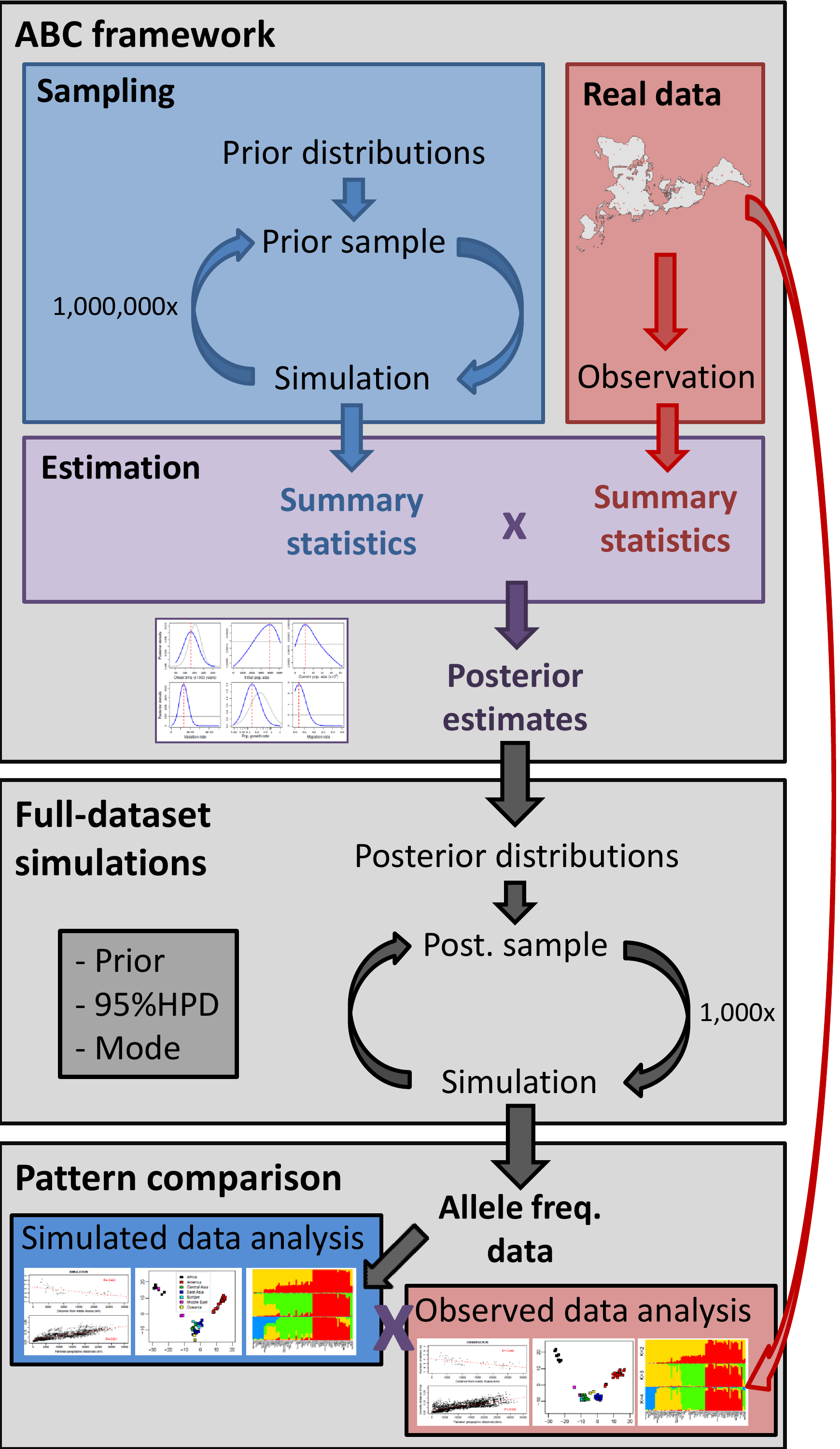

Supplement: S1 Fig — ABC framework shows the basic structure of an ABC analysis focused in parameter estimation. Full-dataset simulations represents the following step in which simulations were run based on the estimations above and for which complete allele frequency data was retained. In Pattern comparison, further analyses were run in order to compare simulations and observations in way they produce results for IBD regression analysis, PCA and STRUCTURE. (TIF) [file pone.0192460.s001.tif]

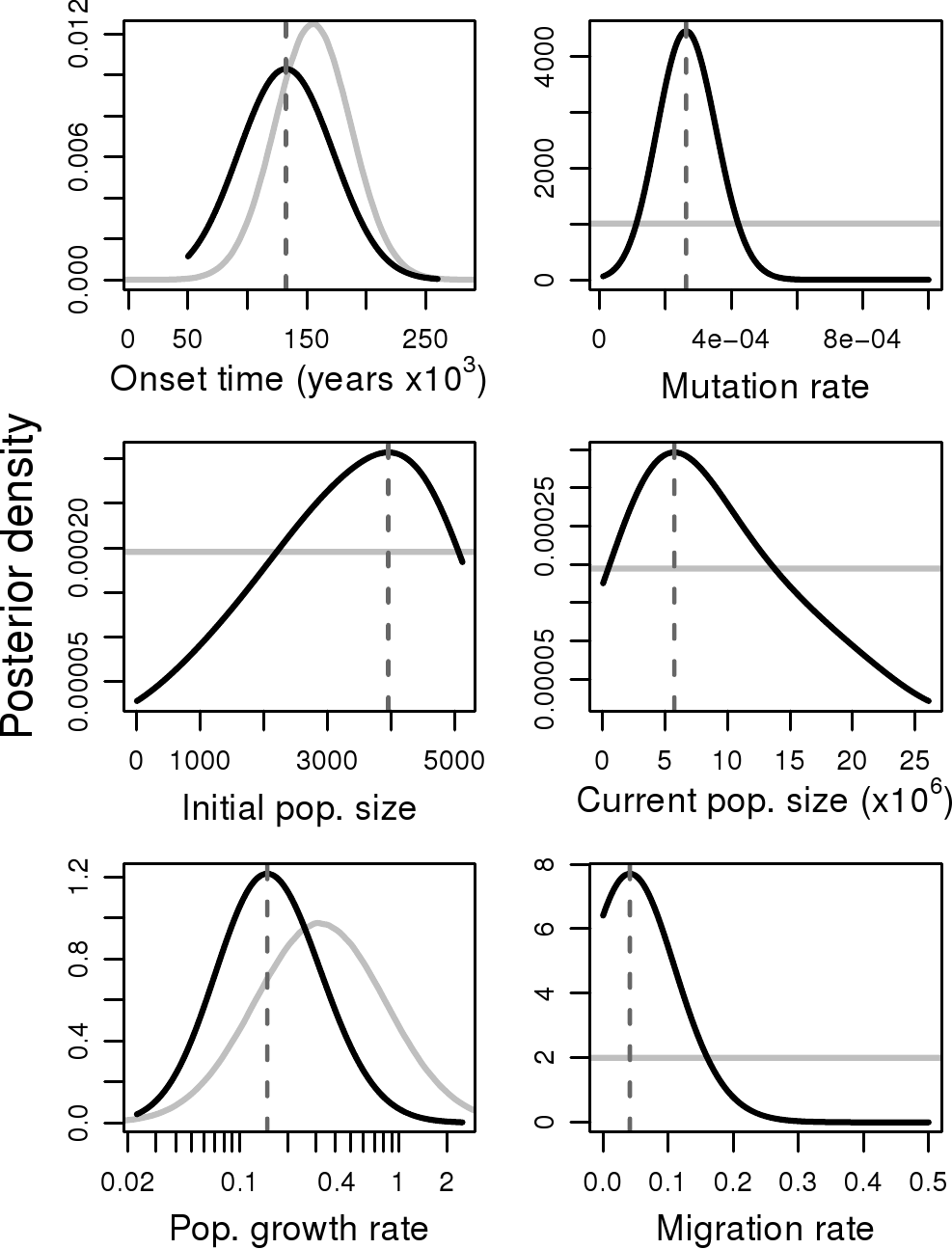

Supplement: S3 Fig — Gray lines represent the prior distributions; black lines, the posteriors; the gray dashed vertical lines, the modes for the posteriors (point estimates). The estimations were carried out on 5,000 out of ~1 million simulations which were the closest to the observations in six pattern statistics (see material and methods for details). (TIF) [file pone.0192460.s003.tif]

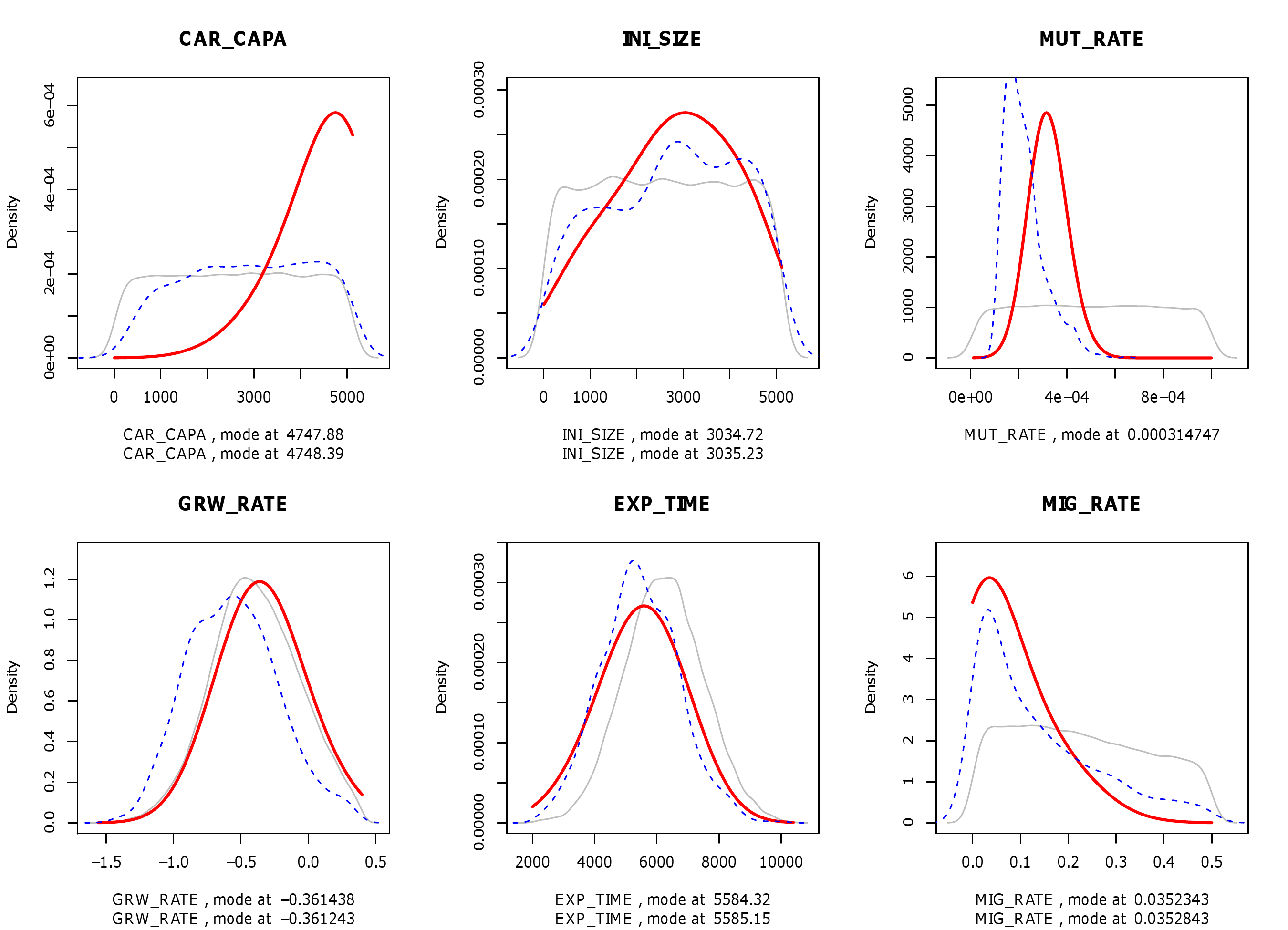

Supplement: S4 Fig — Gray lines represent the realized priors; blue dashed lines represent the distribution of the parameter values in the retained simulations; red lines represent the posterior distributions. The PLS calculation was conducted on a set of 2,485 statistics comprising number of alleles (A) and gene diversity (Hs) per patch and all pairwise FST comparisons between patches. CAR_CAPA stands for current population size; INI_SIZE, initial population size; MUT_RATE, mutation rate; GRW_RATE, population growth rate; EXP_TIME, time of onset of the expansion; MIG_RATE, migration rate. Below each panel, the values for the mode (point estimates) are given for every parameter. (TIF) [file pone.0192460.s004.tif]

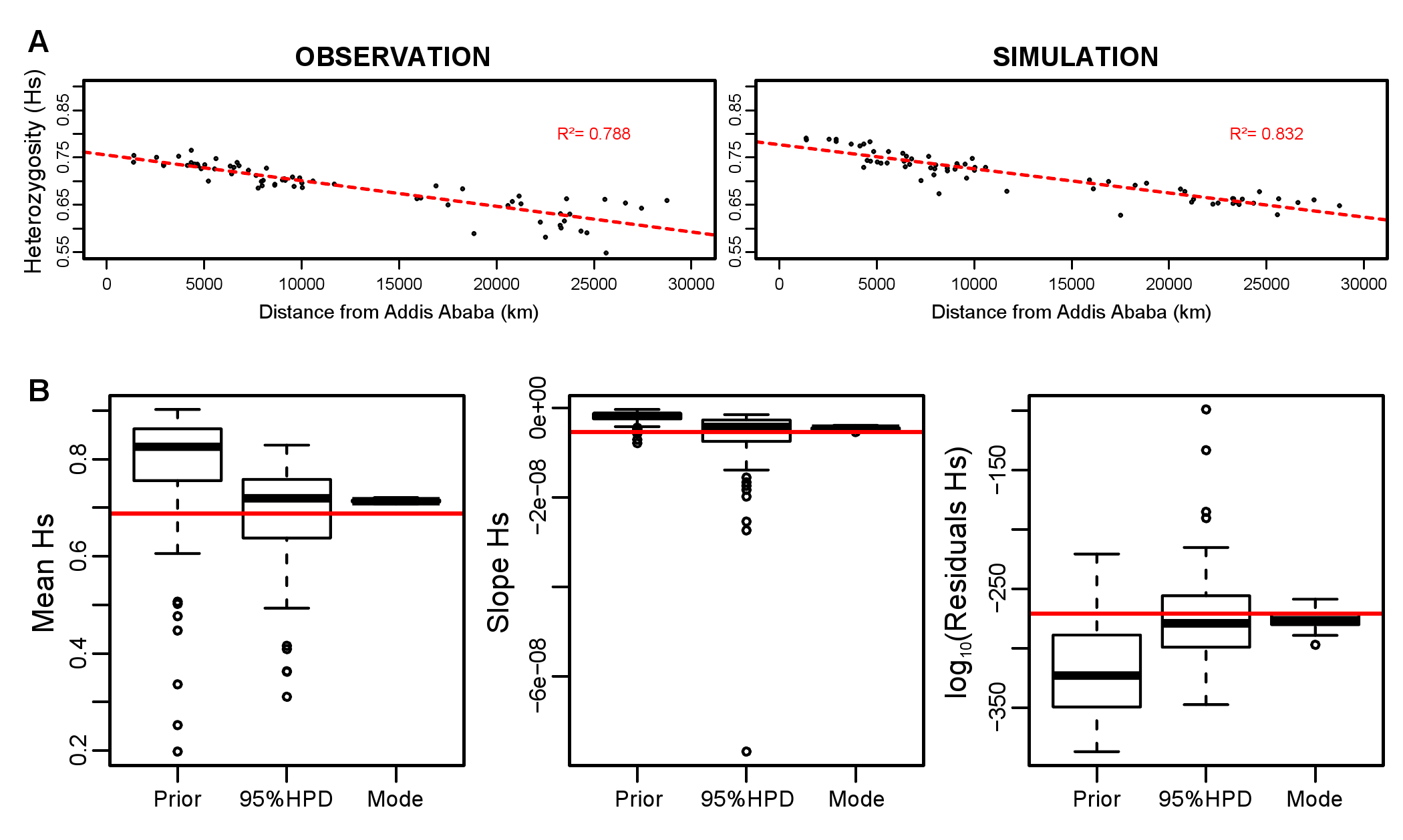

Supplement: S5 Fig — A, comparison of the patterns generated for the cline in heterozigosity between observation and a simulation based on the point estimates. B, convergence of different pattern statistics related to the heterozigosity cline across different samplings from prior or posterior. (TIF) [file pone.0192460.s005.tif]

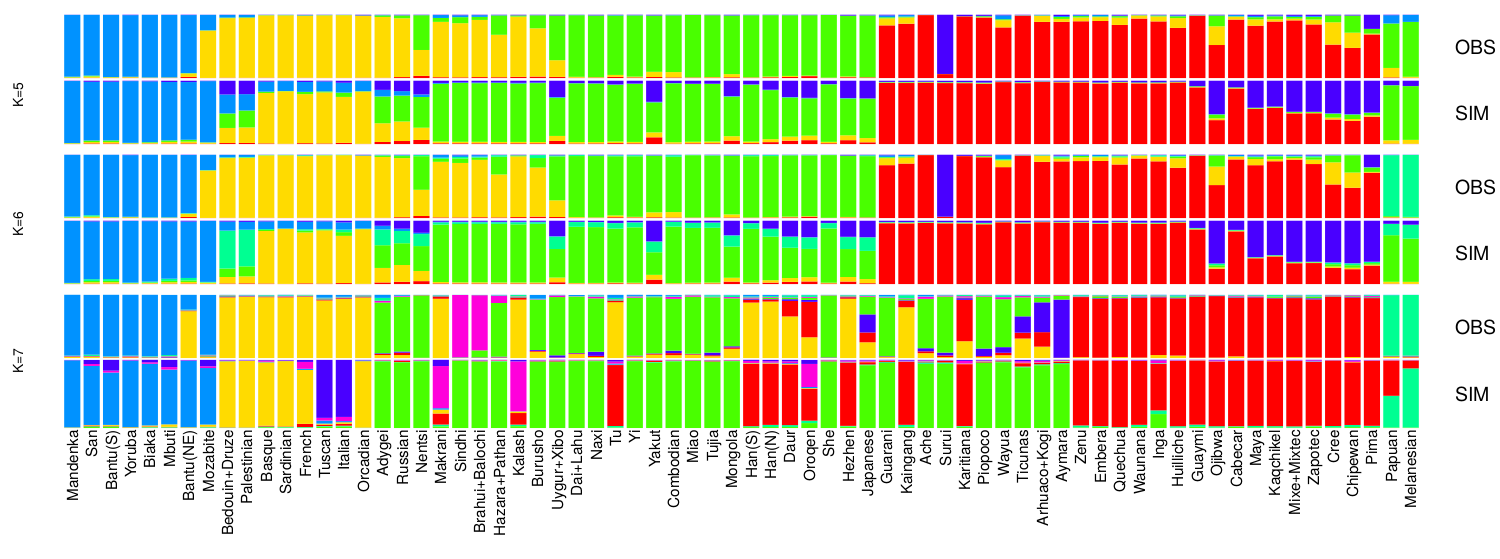

Supplement: S6 Fig — Vertical bars represent the 70 populations as used in the simulations and the colors code for the proportion of each inferred ancestry group (K = 5, 6 and 7). One can observe that particular populations become highlighted in the observations (Suruí with K = 5, Oceanians with K = 6); while, in the simulations, many populations begin to show admixed compositions. (TIF) [file pone.0192460.s006.tif]

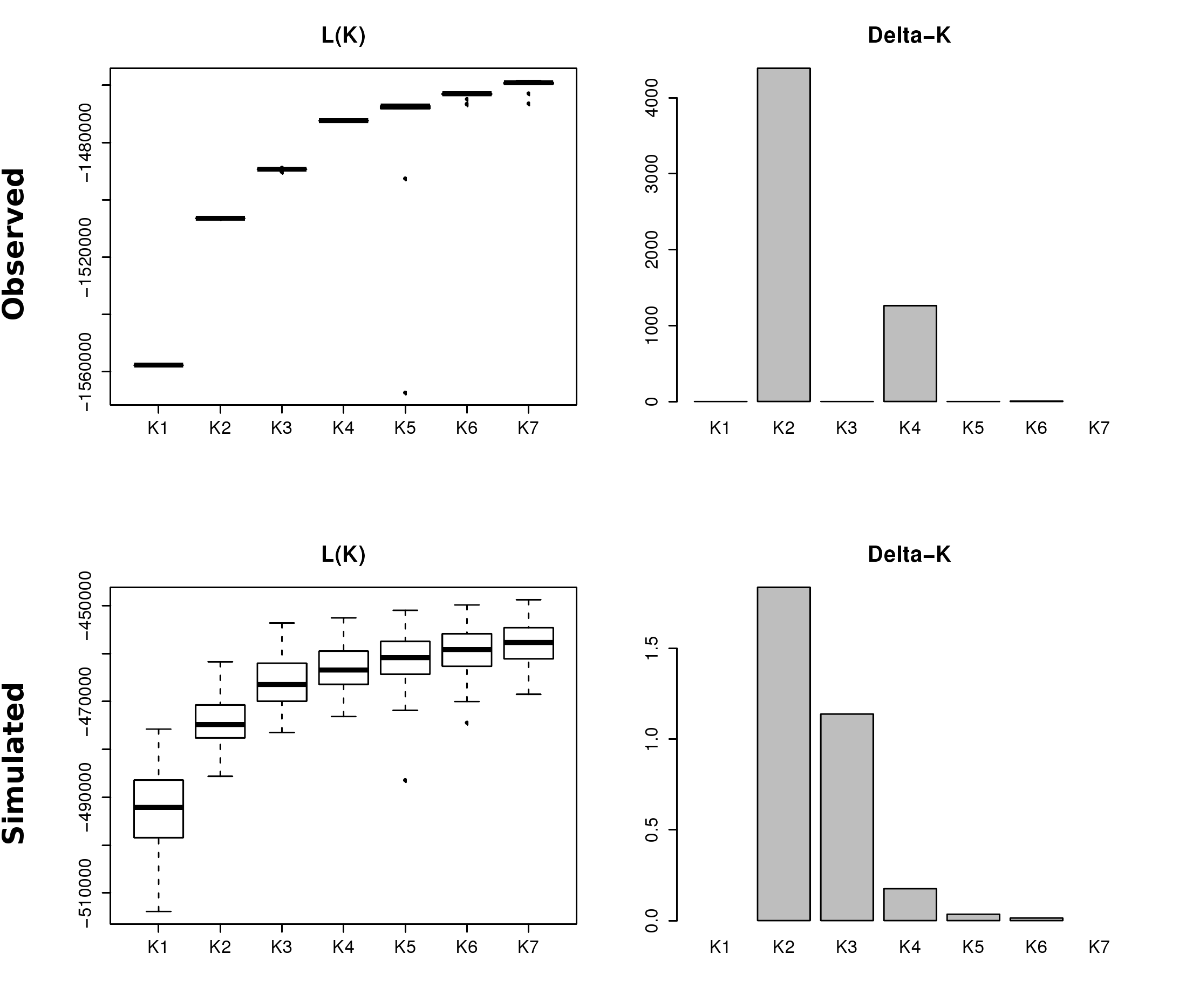

Supplement: S7 Fig — The figure contains the results obtained both for observations (Observed) and simulations (Simulated). L(K) is the direct assessment of likelihood for each number of groups. Delta-K is the estimate based on Evanno et al.’s 2005 approach. (TIF) [file pone.0192460.s007.tif]
