# Supplementary material for: Complex genetic patterns in human arise from a simple range-expansion model over continental landmasses
Source: PLoS One. 2018 Feb 21;13(2):e0192460. doi: 10.1371/journal.pone.0192460 (PMC5821356; doi:10.1371/journal.pone.0192460)
Supplement: S1 Table — Populations marked with “a” were merged together due to their geographical proximity (less than 160km apart) and were considered to inhabit the same deme in the simulations and also in the analyses applied to the read dataset. Populations marked with “b” were removed from the pattern statistics calculations: They were either known exceptions to the general patterns found in the continent (Aché), or were sampled in the vicinity of other populations, on the edges of their original distributions. For these, we kept the populations with the larger sample sizes and these were the Karitiana (as opposed to the Suruí) and Guarani (as opposed to the Kaingang). (PDF) [file pone.0192460.s008.pdf]

Continent	Population	Number of individuals
Africa	Bantu North-Eastern Africa	12
Africa	Bantu Southern Africa	8
Africa	Biaka Pygmies	32
Africa	Mandenka	24
Africa	Mbuti Pygmies	15
Africa	San	7
Africa	Yoruba	25
America	Ache[b]	19
America	Arhuaco & Kogi[a]	34
America	Aymara	18
America	Cabecar	20
America	Chipewan	29
America	Cree	18
America	Embera	11
America	Guarani	10
America	Guaymi	18
America	Huilliche	20
America	Inga	17
America	Kaingang[b]	7
America	Kaqchikel	12
America	Karitiana	24
America	Maya	25
America	Mixe & Mixtec[a]	40
America	Ojibwa	20
America	Pima	25
America	Piopoco	13
America	Quechua	20
America	Surui[b]	21
America	Ticuna-Arara & Ticuna-Tarapaca[a]	35
America	Waunana	20
America	Wayua	17
America	Zapotec	19
America	Zenu	18
South-Asia	Brahui & Balochi[a]	50
South-Asia	Burusho	25
South-Asia	Hazara & Pathan[a]	48
South-Asia	Kalash	25
South-Asia	Makrani	25
South-Asia	Sindhi	25
South-Asia	Uygur & Xibo[a]	19
East-Asia	Combodian	11
East-Asia	Dai & Lahu[a]	20

East-Asia	Daur	10
East-Asia	Han Central China	34
East-Asia	Han Northern China	10
East-Asia	Hezhen	9
East-Asia	Japanese	29
East-Asia	Miao	10
East-Asia	Mongola	10
East-Asia	Naxi	10
East-Asia	Oroqen	10
East-Asia	She	10
East-Asia	Tu	10
East-Asia	Tujia	10
East-Asia	Yakut	25
East-Asia	Yi	10
Europe	Adygei	17
Europe	Basque	24
Europe	Bedouin & Druze[a]	95
Europe	French	29
Europe	Italian	13
Europe	Mozabite	30
Europe	Orcadian	16
Europe	Palestinian	51
Europe	Russian	25
Europe	Sardinian	28
Europe	Tundra Nentsi	14
Europe	Tuscan	8
Oceania	Melanesian	19
Oceania	Papuan	17
