# Supplementary material for: Complex genetic patterns in human arise from a simple range-expansion model over continental landmasses
Source: PLoS One. 2018 Feb 21;13(2):e0192460. doi: 10.1371/journal.pone.0192460 (PMC5821356; doi:10.1371/journal.pone.0192460)
Supplement: S2 Table — (PDF) [file pone.0192460.s009.pdf]

Parameter	Abbreviation	Distribution	Min.	Max.	Mean	S.D.
Initial Population Size	N_i	Uniform	2	5120	-	-
Carrying Capacity	K	Uniform	2	5120	-	-
Growth rate	r	Lognormal	0.01	2.5	0.5	0.6
Migration rate	m	Uniform	0	0.5	-	-
Time of the onset	T	Normal	2000	10400	6200	1280
Mutation rate	μ	Uniform	1.00E-05	1.00E-03	-	-
